# Sequence of joint tissue inflammation during rheumatoid arthritis development

**DOI:** 10.1186/s13075-018-1756-z

**Published:** 2018-11-21

**Authors:** R. M. ten Brinck, H. W. van Steenbergen, A. H. M. van der Helm–van Mil

**Affiliations:** 1 Department of Rheumatology C1-R, Leiden University Medical Centre, PO Box 9600, Leiden, 2300RC the Netherlands; 2 Department of Rheumatology, Erasmus Medical Centre, Rotterdam, The Netherlands

**Keywords:** Rheumatoid arthritis, Inflammation, Imaging

## Abstract

**Objective:**

Subclinical joint inflammation in patients with arthralgia is predictive for progression to rheumatoid arthritis (RA). However, the time course of progression for bone marrow edema (osteitis), synovitis, and/or tenosynovitis is unsettled. This longitudinal study assessed the course of magnetic resonance imaging (MRI)-detected subclinical joint inflammation during progression to RA.

**Methods:**

Patients that progressed from clinically suspect arthralgia (CSA) to RA underwent 1.5-T MRI of the metacarpophalangeal (MCP), wrist, and metatarsophalangeal (MTP) joints at presentation with arthralgia and at first identification of synovitis assessed through physical examination (*n* = 31). MRIs were evaluated for osteitis, synovitis, tenosynovitis, and erosions by two readers, blinded for clinical data and order in time. To estimate changes in MRI scores between the asymptomatic state and CSA onset, scores of MRI features at CSA baseline were compared with scores from age-matched symptom-free persons.

**Results:**

At presentation with CSA, synovitis and tenosynovitis scores were higher than scores from age-matched symptom-free persons (*p* = 0.004 and *p* = 0.001, respectively). Anti-citrullinated protein antibody (ACPA)-positive arthralgia patients also had increased osteitis scores (*p* = 0.04). Median duration between presentation with arthralgia and RA development was 17 weeks. During progression to RA, synovitis and osteitis increased significantly (*p* = 0.001 and *p* = 0.036, respectively) in contrast to tenosynovitis and erosion scores. This pattern was similar in both ACPA subsets, although statistical significance was reached for synovitis and osteitis in ACPA-negative but not ACPA-positive RA.

**Conclusion:**

Increased tenosynovitis and synovitis scores at CSA onset and the increase in synovitis and osteitis during progression to RA suggest an ‘outside-in’ temporal relationship of arthritis development, in particular for ACPA-negative RA. For ACPA-positive RA, further studies are needed.

**Electronic supplementary material:**

The online version of this article (10.1186/s13075-018-1756-z) contains supplementary material, which is available to authorized users.

## Background

Rheumatoid arthritis (RA) can be diagnosed at the time patients present with clinically detectable inflammatory arthritis (swollen joints). It is known that immunological alterations are present long before that [1]. For instance, magnetic resonance imaging (MRI)-detected subclinical inflammation is present weeks to months before arthritis becomes clinically apparent in patients presenting with clinically suspect arthralgia (CSA) and has been shown to be predictive for RA development [2]. Nonetheless, a long-standing question is where inflammation starts in the joint, or in what order the different tissues of the joints (synovium, tenosynovium, and bone) become inflamed during the development of RA.

Several hypotheses on the chronology of arthritis development prevail. Firstly, it has been postulated that synovitis is an initial process that is succeeded by bone involvement. This is the traditional ‘outside-in hypothesis’, presuming that inflammation of the synovium precedes bone marrow edema (or osteitis) [3–7]. Alternatively, it has been suggested that RA is a primary bone marrow disease which subsequently encroaches upon the synovial membrane; this is the ‘inside-out hypothesis’, with osteitis preceding synovitis, a hypothesis that has become popular after imaging and histological studies revealed the presence of osteitis at locations with MRI-detected osteitis in patients with RA [4, 7, 8]. It has also been hypothesized that these processes could emerge and progress simultaneously with microscopic bone canals allowing transduction of inflammation from the outside (synovium) to the inside (bone marrow) and vice versa [4, 7, 9]. Finally, mouse models of arthritis development have shown that tenosynovitis was the initial preclinical change [10]. Extrapolation to humans would suggest that tenosynovitis rather than synovitis or osteitis is the primary feature of joint inflammation [10]. For the development of bone erosions (instead of the development of clinically apparent inflammatory arthritis) similar hypotheses have been postulated, with the primary inflammation located inside [11, 12] or outside the bone [13], respectively. Altogether, temporal relationships are yet unknown and can be discovered by longitudinal imaging studies that start in pre-arthritis phases of the disease.

To address the question about the chronological order in which the different tissues of the joints become inflamed, this longitudinal MRI study investigated the course of MRI-detected subclinical joint inflammation (synovitis, tenosynovitis, osteitis) and erosions in patients that presented with arthralgia and progressed to RA. MRIs were performed longitudinally at presentation with arthralgia and at development of RA. Although no MRIs were made in the asymptomatic phase of these patients, the MRI data obtained at presentation with arthralgia were compared with data obtained in age-matched symptom-free persons to estimate the course of inflammation before presentation with arthralgia. Finally, as anti-citrullinated protein antibody (ACPA)-positive and ACPA-negative RA are considered as different disease subsets with differences in the underlying pathophysiology, analyses were stratified for ACPA-positive and ACPA-negative RA to explore if there are differences in the chronological order in which different articular tissues become inflamed.

## Patients and methods

### Patients

We longitudinally followed patients that presented with CSA between April 2012 and September 2016. The Leiden CSA cohort consecutively included patients that presented at the rheumatology outpatient clinic of the Leiden University Medical Centre without clinically evident arthritis, but with recent-onset (< 1 year) arthralgia of small joints that was considered at risk for RA by their rheumatologists based on the clinical presentation [14]. A detailed description of the inclusion and exclusion criteria of the Leiden CSA cohort and the study protocol are described in [15]. Absence of clinical arthritis at baseline was ascertained through physical examination by an experienced rheumatologist.

Regular follow-up visits in the cohort were planned at 4, 12, and 24 months. If necessary (for instance, when the patient experienced more symptoms or noticed a swollen joint), patients were seen in between scheduled visits by their rheumatologist. Hence, logistics were arranged such that patients in this cohort had very easy access to rheumatology care, and clinically evident inflammatory arthritis was identified at the first opportunity. The outcome was clinically apparent arthritis, identified at joint examination by an experienced rheumatologist. Previous studies revealed that ~ 20% of patients that presented with CSA progressed to clinically apparent inflammatory arthritis [2].

**Fig. 1:**
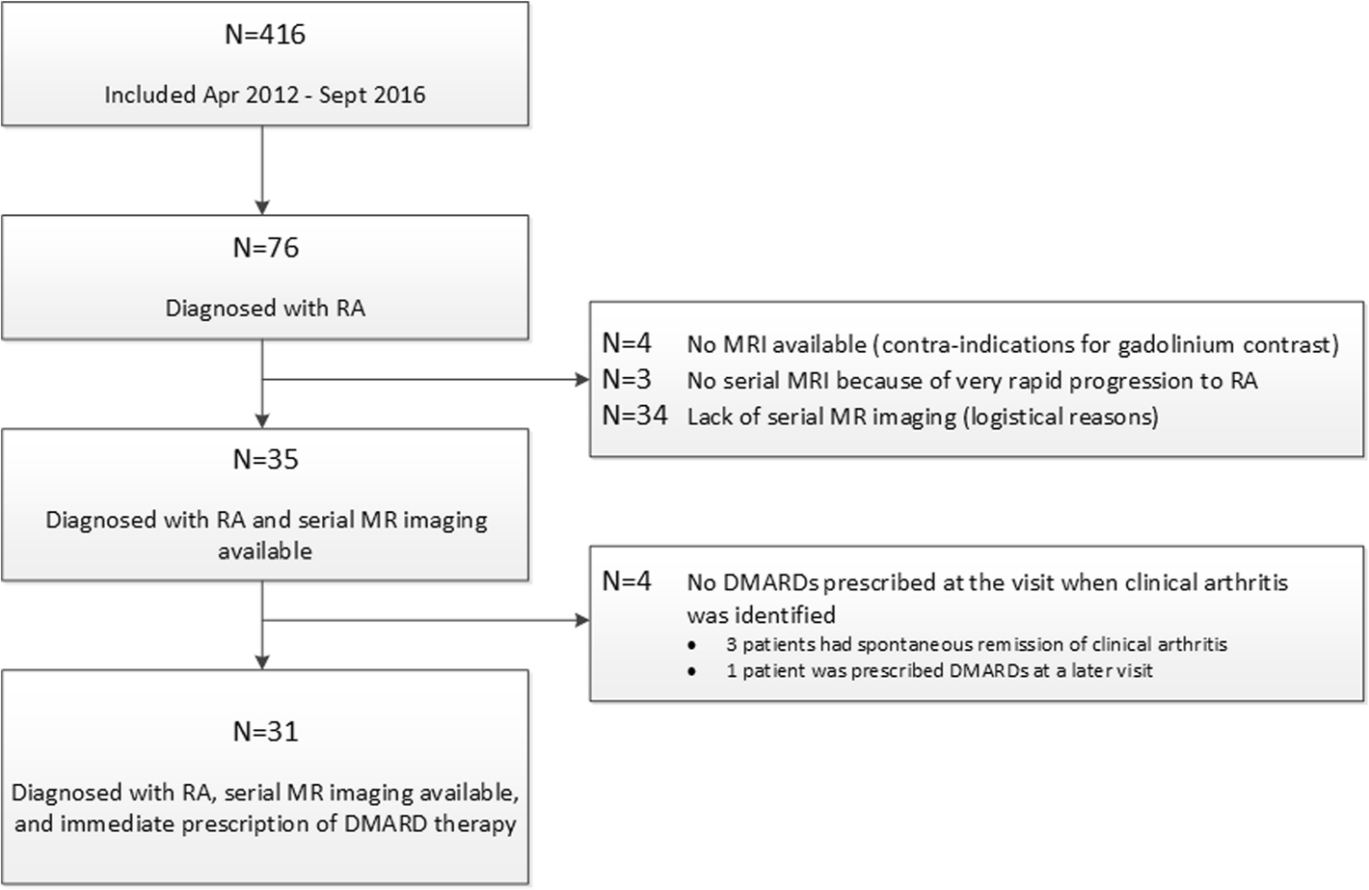
Flowchart of selection of rheumatoid arthritis (RA) patients studied from the total CSA cohort. Patient characteristics of the different groups are provided in Additional file 1 (Table S2). DMARD disease-modifying antirheumatic drug, MR magnetic resonance, MRI magnetic resonance imaging

For this study we selected the patients included in the CSA cohort that developed clinically apparent RA. A flowchart is provided in Fig. 1. From a total of 416 patients included in the CSA cohort during the studied period, 76 were diagnosed with RA. Serial MRI was performed at baseline and at arthritis development (but before disease-modifying antirheumatic drugs (DMARDs) were started). Of the 76 RA patients, serial MRIs were available in 35 patients. In 41 patients serial imaging was not available because four patients had contraindications for gadolinium contrast, three patients progressed to RA in a very short period of time, and 34 patients were lacking serial MRI due to logistical reasons.

Thirty-five patients were diagnosed with RA and had serial MRI available. Four out of these 35 patients did not receive a prescription for a DMARD at the visit when clinical arthritis was identified (three patients subsequently had resolution of arthritis, one patient received a prescription at the next visit). Thus, in total 31 patients with a clinical diagnosis of RA and immediate prescription of DMARDs were studied. Notably, fulfilment of the 2010 classification criteria was not required to define RA since ACPA-negative patients require > 10 involved joints to fulfil these criteria [16] and this can be hampered by DMARD initiation. Nonetheless, despite this theoretical threshold, 21 patients (68%) fulfilled the 2010 criteria for RA at arthritis development. It is important to note that DMARDs (including corticosteroids) were not prescribed in the phase of arthralgia.

### Symptom-free persons

Serial MRIs were not made in the period preceding presentation with CSA. To infer the course of MRI-detected subclinical inflammation over time before the phase of CSA, the MRI data from the 31 CSA patients were compared with data from symptom-free persons [17]. Symptom-free persons were matched based on age in a 1:2 ratio. These 62 symptom-free persons had no history of inflammatory rheumatic diseases, no joint symptoms during the previous month, and no evidence of synovitis at physical examination. The persons were retrieved from the general population; the recruitment procedure is described elsewhere [17]. Since gender (*p* = 0.10), increased body mass index (BMI) (*p* = 0.59) [18], and smoking (*p* = 0.21) had no effect on MRI-detected inflammation, matching was not performed on these factors.

### MRI

Unilateral MRIs of the wrist, metacarpophalangeal (MCP) joints 2–5, and metatarsophalangeal (MTP) joints 1–5 were performed at presentation with CSA (on the most painful side) and at first presentation with clinical synovitis (the same side as scanned at baseline). A total of 18 tenosynovitis locations were scored in each patient: 10 at the wrist, including 6 extensor compartments and 4 regions on the volar side (the flexor digitorum profundus and flexor digitorum superficialis, the flexor pollicis longus, the flexor carpi ulnaris, and the flexor carpi radialis), and 8 locations at MCP joints 2–5 (paired flexor tendons and extensor tendons of the fingers). An ONI MSK Extreme 1.5-T MRI scanner (GE Healthcare, Wisconsin, USA) was used as described previously [2] and in Additional file 1 (Supplementary methods). All patients were instructed not to use nonsteroidal anti-inflammatory drugs (NSAIDs) 24 h prior to MRI, with only seven patients (23%) reporting the daily use of NSAIDs at baseline. The serial MRIs were scored blinded for clinical data and order in time by two readers for osteitis, erosions, and synovitis as described in [19] and tenosynovitis as described in [20]. The mean scores of two readers were studied. Additional information on the scoring is provided in Additional file 1 (Supplementary methods). Three readers contributed to the scoring of the MRIs. Within-reader intraclass correlation coefficients (ICCs) and between-reader ICCs were all > 0.90 and are also presented in Additional file 1 (Table S1). Results of MRI were not shared with the treating rheumatologists.

### Analyses

Paired *t* tests were used. Longitudinal modeling was performed using generalized estimating equations (GEE); the scores of the different inflammatory features and erosions were studied over time. The GEE models, utilizing an unstructured correlation matrix, corrected for the influence of age, gender, and time to progression to RA and baseline score per feature. Residuals of GEE modeling were checked for normal distribution. Statistical analyses were performed using the Statistical Package for the Social Sciences (SPSS, version 23.0). *P* values < 0.05 were considered significant.

## Results

### Patients

Characteristics of the 31 RA patients studied were similar to the total group of 76 patients diagnosed with RA, except for a lower frequency of ACPA positivity in the studied group (Additional file 1: Table S2). Baseline characteristics of the 31 patients studied are presented in Table 1. The mean age was 44 years, 71% were female, the median tender joint count (68-TJC) was 5 (interquartile range (IQR) 4–10), 42% were rheumatoid factor-positive, and 29% were ACPA-positive. These characteristics are in line with previous research in CSA patients [15]. The median interval between presentation with arthralgia and progression to RA was 17 weeks. The characteristics of the 31 patients at the time of identification of RA are also presented in Table 1. The DMARDs that were initiated after RA development are presented in Additional file 1 (Table S3).

**Table 1:** Characteristics of the RA patients at presentation with clinically suspect arthralgia and at first identification of clinical arthritis and age-matched symptom-free volunteers

	At presentation with arthralgia (*n* = 31)	At presentation with RA (*n* = 31)	Age-matched symptom-free volunteers (*n* = 62)
Age (years), mean (SD)	44 (14)	–	44 (13)
Female sex, *n* (%)	22 (71)	–	38 (61)
Symptom duration (weeks), median (IQR)	17 (9–32)	–	n/a
Presence of morning stiffness ≥ 60 min, *n* (%)	11 (35)	24 (77)	0 (0)
68-TJC, median (IQR)	5 (4–10)	7 (5–12)	0 (0–0)
66-SJC, median (IQR)	0 (0–0)	2 (1–5)	0 (0–0)
Increased CRP (≥ 5 mg/L), *n* (%)	6 (19)	7 (29)	n/a
Autoantibody status			
IgM-RF-positive (≥ 3.5 IU/mL), *n* (%)	13 (42)	13 (42)	n/a
ACPA-positive (≥ 7 U/mL), *n* (%)	9 (29)	9 (29)	n/a
Any antibody-positive (either RF or ACPA)	16 (52)	16 (52)	n/a

*ACPA* anti-citrullinated peptide antibody, *CRP* C-reactive protein, *IgM-RF* immunoglobulin M rheumatoid factor, *IQR* interquartile range, *n/a* not applicable or not assessed, *RA* rheumatoid arthritis, *RF* rheumatoid factor, *SD* standard deviation, *SJC* swollen joint count, *TJC* tender joint count

### MRI data at presentation with arthralgia compared with that of age-matched symptom-free controls

**Fig. 2:**
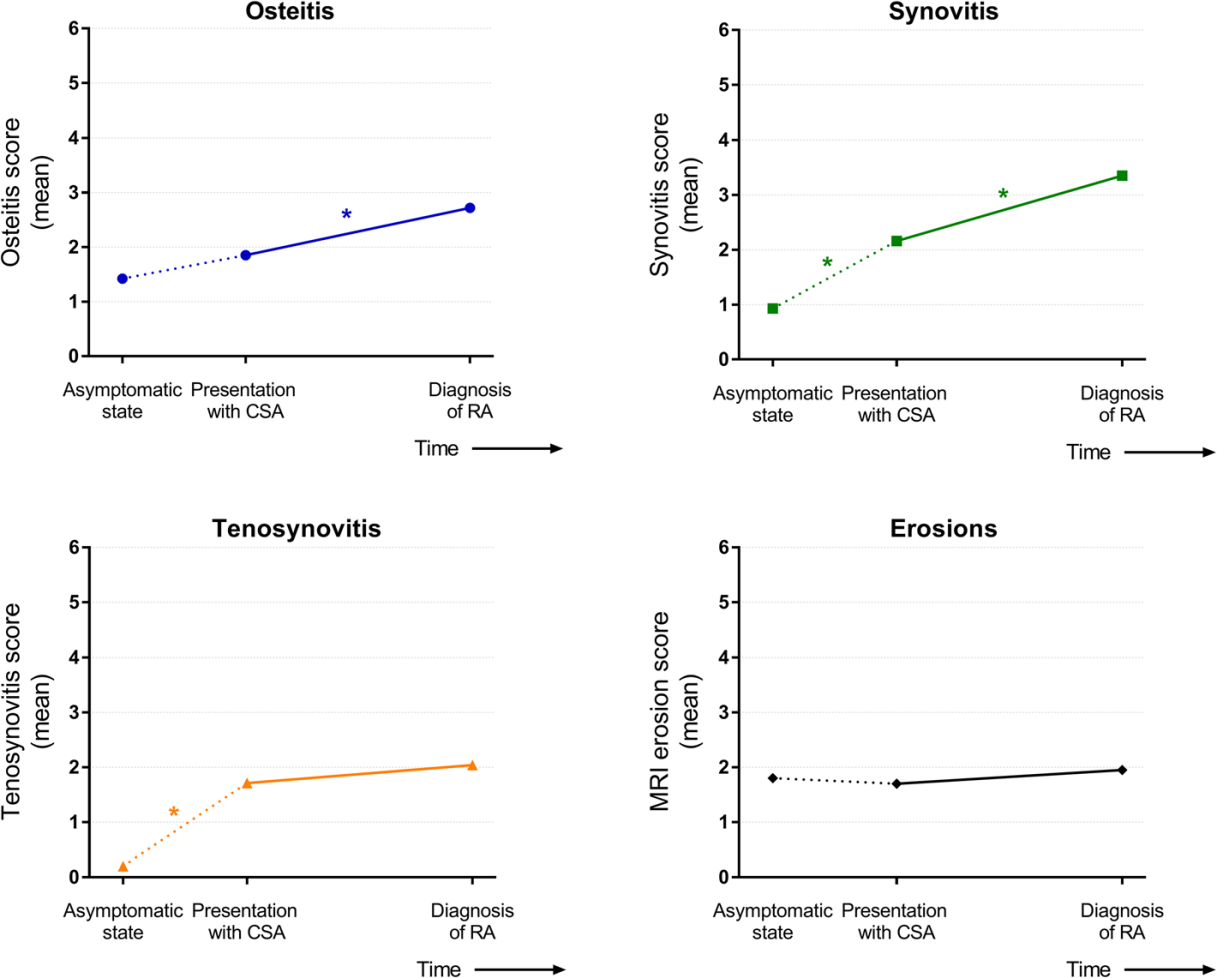
Magnetic resonance imaging (MRI) features of joint inflammation and erosions in patients that developed rheumatoid arthritis (RA). MRI was performed at presentation with arthralgia and at diagnosis of RA. MRIs were not made in the asymptomatic phase but the course of local inflammation before presentation with arthralgia was estimated by comparisons with age-matched symptom-free persons (1:2 ratio to patients). Since these data were not measured longitudinally, data are presented in dashed lines. *p < 0.05. CSA clinically suspect arthralgia

Compared with age-matched symptom-free persons from the general population, CSA patients had increased tenosynovitis (mean 1.7 versus 0.27; *p* < 0.001) and synovitis scores (2.2 versus 0.93; *p* < 0.001; Fig. 2). Osteitis scores (1.9 versus 1.4; *p* = 0.35) and erosion scores (1.7 versus 1.8; *p* = 0.53) were not significantly elevated (Fig. 2) at presentation with arthralgia.

### Inflammation increased during progression from arthralgia to rheumatoid arthritis

During progression from arthralgia to RA, the mean osteitis score increased from 1.9 to 2.7 (*p* = 0.036), and the mean synovitis score from 2.2 to 3.4 (*p* = 0.001; Fig. 2). Tenosynovitis and erosion scores did not increase significantly (mean 1.7 to 2.0, *p* = 0.35, and 1.7 to 1.9, *p* = 0.092, respectively; Fig. 2). Next, GEE models were constructed, allowing longitudinal data comparisons of MRI inflammatory scores. These models corrected for gender, age at inclusion, time interval to RA, and the score of each feature (e.g., osteitis) at presentation with CSA. GEE modeling demonstrated that only synovitis scores (β = 1.0; *p* = 0.004) were significantly higher at the time of RA development than at presentation with arthralgia.

### Stratification for ACPA status

Finally, we explored if results were different in ACPA-positive and ACPA-negative RA patients, although absolute numbers were small (*n* = 9 and *n* = 22, respectively). ACPA-positive patients had higher osteitis scores (*p* = 0.04), synovitis scores (*p* < 0.001), and tenosynovitis scores (*p* < 0.001) at presentation with arthralgia compared with age-matched symptom-free persons. ACPA-negative patients had higher synovitis scores (*p* = 0.02) and tenosynovitis scores (*p* = 0.001), but the osteitis score was not different (*p* = 0.24). Erosion scores were not different from age-matched symptom-free persons in both ACPA-positive and ACPA-negative patients at presentation with arthralgia.

**Fig. 3:**
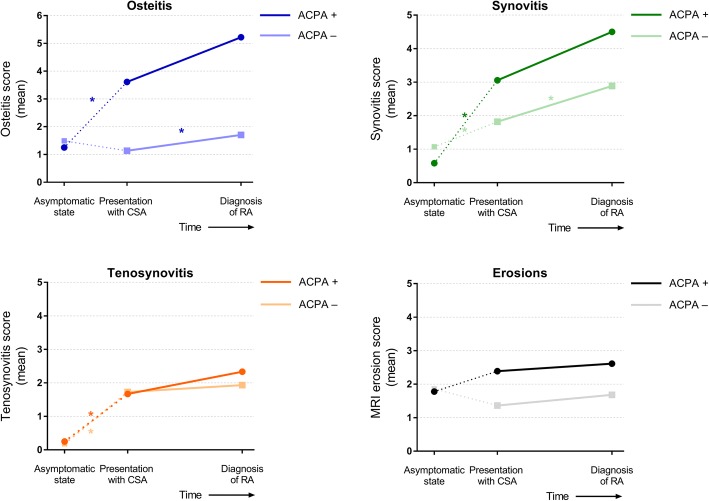
Magnetic resonance imaging (MRI) features of joint inflammation and erosions in patients that developed rheumatoid arthritis (RA) stratified for anti-citrullinated protein antibody (ACPA) status. MRI was performed at presentation with arthralgia and at diagnosis of RA. MRIs were not made in the asymptomatic phase but the course of local inflammation before presentation with arthralgia was estimated by comparisons with age-matched symptom-free persons (1:2 ratio to patients). Since these data were not measured longitudinally, data are presented in dashed lines. *p < 0.05 by paired *t* test. CSA clinically suspect arthralgia

Over time, during progression from arthralgia to RA, ACPA status did not change for any of the patients. In ACPA-negative patients, osteitis and synovitis score increased significantly (1.1 to 1.7, *p* = 0.03, and 1.8 to 2.9, *p* = 0.003, respectively) during progression from arthralgia to RA, whereas tenosynovitis and erosion scores remained stable (Fig. 3). In ACPA-positive patients, no statistical significance was obtained but osteitis scores increased from 3.6 to 5.2 (*p* = 0.22) and synovitis from 3.1 to 4.5 (*p* = 0.13). Tenosynovitis scores were 1.7 and 2.3 (*p* = 0.32) and erosion scores were 2.4 and 2.6 at presentation with arthralgia and RA, respectively (*p* = 0.62; Fig. 3).

Finally, analyses were repeated for autoantibody-positive (positive for either rheumatoid factor and/or ACPA; *n* = 16) and autoantibody-negative patients (*n* = 15). Similar results were obtained as for ACPA-positive and ACPA-negative RA patients as described above (data not shown).

## Discussion

To increase our understanding of the chronological order of joint inflammation during the development of RA, serial MRIs were made in RA patients at the time of presentation with arthralgia and at first identification of RA. This revealed that synovitis and osteitis increased during the symptomatic pre-arthritis phase. In addition, data on MRI-detected subclinical inflammation obtained at presentation with arthralgia were compared with data from age-matched symptom-free persons. This showed that synovitis and tenosynovitis scores were higher in patients with arthralgia, suggesting that these features had already increased at presentation with arthralgia. The erosion scores did not increase, neither in the asymptomatic nor during the symptomatic phase preceding the identification of RA. Together, these results suggest that tenosynovitis and synovitis are the earliest inflammatory features. Subsequently, synovitis and osteitis increased over time in the symptomatic pre-arthritis phase, whereas erosions did not increase before RA had become apparent. This implies that inflammation mainly starts outside the bone (fitting the outside-in hypothesis), after which osteitis develops, which puts the joint at risk for structural damage development in the phase of clinically evident RA (if left insufficiently treated).

Stratification for ACPA status showed that synovitis and osteitis progressed similarly during the symptomatic pre-arthritis phase in both ACPA subsets, but that ACPA-positive arthralgia patients already had higher osteitis scores at presentation with arthralgia. This could be explained by a longer symptom duration of ACPA-positive patients at presentation with arthralgia: median symptom duration at baseline was 22 weeks in ACPA-positive CSA patients and 15 weeks in ACPA-negative CSA patients (Additional file 1: Table S3). As established previously, ACPA-positive RA has a more gradual onset of symptoms [21]. ACPA-positive patients may therefore have presented in a slightly later phase of the disease. Alternatively, this finding could also imply a different chronology of joint inflammation in ACPA-negative and ACPA-positive RA, with osteitis being one of the first inflammatory features in ACPA-positive RA. This could be in line with the associations of autoantibody status and osteitis scores observed in the phase of classifiable RA [22].

There are several limitations to this study. One limitation is that the analyses were performed on the sum of synovitis, osteitis, and tenosynovitis scores observed in 310 joints, 1023 bones, and 868 tendons. Whilst it would be interesting to perform similar analyses at the individual joint/bone/tendon level, this was not done because of low numbers of joints/bones/tendons with subclinical inflammation. Larger studies are needed to this end.

Another limitation is the sample size. Although all patients that developed RA from a total cohort of consecutive CSA patients (with serial MRI available) were studied, the absolute number (*n* = 31) is rather small. Future longitudinal imaging studies are therefore required to validate the results. Nonetheless, the present study is the largest longitudinal MRI study in a population of patients that converted from arthralgia to RA that is available to date.

Analyses were stratified for ACPA to explore differences between these subgroups, although the ACPA-positive subgroup especially was rather small. This may be explained by more intramuscular corticosteroid injections in ACPA-positive patients, preventing the performance of an MRI before DMARD initiation. Larger future studies, especially in ACPA-positive convertors, are needed.

Another limitation is that MRIs were made at first presentation with CSA and at development of RA, but no scans were made in between. Furthermore, patients were included at first presentation with clinically suspect arthralgia and were not scanned in an asymptomatic phase. Although some information on the chronology of joint inflammation in this disease phase was obtained by comparison of data obtained in age-matched symptom-free persons, this analysis was cross-sectional in nature and therefore less reliable than longitudinally collected data. Hence, future longitudinal imaging studies would be still highly relevant to further increase our understanding of RA development.

In our study, T1-weighted fat-suppressed sequences were obtained. These were previously demonstrated to have a strong correlation with T2-weighted fat-suppressed sequences in three studies [23–25]. Furthermore, persistent osteitis was strongly associated with erosive progression using these sequences [11]. Taken together, these findings demonstrate that osteitis is an established risk factor for the development of articular bone erosions regardless of the acquired sequence. The lack of a significant increase in osteitis scores between asymptomatic persons and arthralgia patients is more likely to be caused by the (early) disease stage in which the patients were assessed rather than a difference due to the scanning protocol. Nevertheless, replication of our results using T2-weighted fat-suppressed sequences is warranted.

Future serial imaging studies performed in the asymptomatic phase until development of RA would be useful to further elucidate the chronology in which the different articular tissues become inflamed. However, this cannot be easily accomplished as the 5-year positive predictive value of ACPA in the general population is approximately 5% [26, 27]. Consequently, 20 symptom-free ACPA-positive persons would need to be followed for several years with serial MRIs to acquire longitudinal data of one RA patient. Studying 30 ACPA-positive RA patients already in the asymptomatic phase would require serial imaging in ~ 600 ACPA-positive symptom-free persons during several years of follow-up. Hence, although challenging, studying temporal relationships of inflammatory features during progression from the asymptomatic to the symptomatic pre-arthritis phase is a subject for follow-up research.

Animal studies have demonstrated that tenosynovitis is the earliest inflammatory joint feature in murine models [10]. Studies in CSA patients have also revealed that, of all three inflammatory features, tenosynovitis is the strongest predictor for RA development [2]. The current data, showing that tenosynovitis is a very early phenomenon, denote the importance of inflammation of synovial tendon sheaths in very early phases of RA development.

The erosion score did not increase in the pre-arthritis phase, in contrast with the osteitis score. Previous research has revealed that osteitis that persisted in the early clinical phase of RA was strongly associated with erosion development (odds ratio ~ 60) at the same location [11]. CSA patients in this study had very early access to rheumatologic care in case of symptom deterioration or if they suspected joint swelling. Therefore, we expect that patients were seen very shortly after clinical synovitis had developed. In addition, the median duration to development of RA was relatively short (17 weeks). Both factors may explain why articular bone erosions were not (yet) increased during the studied period. Based on MRI data collected in early arthritis patients [11], we anticipate that the bones with osteitis at RA presentation were at risk for development of erosions, but the time period was too brief for actual erosive progression. This is in line with previous studies showing that MRI-detected erosions predominantly developed in joints with persistent osteitis [11].

Imaging using ultrasound (US) can be used to assess the presence of synovitis and tenosynovitis. Our data, obtained in the earliest disease phases, could suggest that US could suffice for assessment of these features. However, US cannot depict osteitis which may also provide valuable information. Studies in larger patient groups are needed before the present findings can be translated to clinical practice, although the present data contribute to our understanding of clinical arthritis and RA development.

## Conclusions

In summary, the data provided on RA patients suggest an increase of tenosynovitis and synovitis scores before the onset of arthralgia. Our study demonstrated that synovitis and osteitis scores increased during progression from arthralgia to clinical arthritis, suggesting an ‘outside-in’ temporal relationship of arthritis development, particularly in ACPA-negative RA. For ACPA-positive RA further studies are needed.

## Additional file

Additional file 1: **Table S1.** Matrix of ICC scores of three readers who contributed to the data; each MRI was evaluated by two readers. **Table S2.** Comparison of patient characteristics between all patients converting to clinically apparent arthritis, those with serial MRIs, and the final selection of RA patients studied. **Table S3.** Patient characteristics of ACPA-positive and ACPA-negative patients studied at presentation with CSA. **Table S4.** Disease-modifying antirheumatic drugs prescribed when clinical arthritis was identified. **Supplementary methods.** MRI scanning protocol and scoring. (DOCX 42 kb)

## Abbreviations

ACPA: Anti-citrullinated protein antibody
BMI: Body mass index
CSA: Clinically suspect arthralgia
DMARD: Disease-modifying antirheumatic drug
GEE: Generalized estimating equations
ICC: Interclass correlation coefficient
IQR: Interquartile range
MCP: Metacarpophalangeal
MRI: Magnetic resonance imaging
MTP: Metatarsophalangeal
NSAID: Nonsteroidal anti-inflammatory drug
RA: Rheumatoid arthritis
SPSS: Statistical Package for the Social Sciences
TJC: Tender joint count
US: Ultrasound
