# Supplementary material for: Sequence of joint tissue inflammation during rheumatoid arthritis development
Source: Arthritis Res Ther. 2018 Nov 21;20:260. doi: 10.1186/s13075-018-1756-z (PMC6249752; doi:10.1186/s13075-018-1756-z)
Supplement: Supplementary file 1 — Table S1. Matrix of ICC scores of three readers who contributed to the data; each MRI was evaluated by two readers. Table S2. Comparison of patient characteristics between all patients converting to clinically apparent arthritis, those with serial MRIs, and the final selection of RA patients studied. Table S3. Patient characteristics of ACPA-positive and ACPA-negative patients studied at presentation with CSA. Table S4. Disease-modifying antirheumatic drugs prescribed when clinical arthritis was identified. Supplementary methods. MRI scanning protocol and scoring. (DOCX 42 kb) [file 13075_2018_1756_MOESM1_ESM.docx]

# Additional file 1

# Table S1. Matrix of ICC scores of all three readers who contributed to the data; each MRI was evaluated by two readers each.

## Interreader intraclass correlation coefficients

| **Reader** | **1** | **2** | **3** |
| --- | --- | --- | --- |
| **1** | X | 0.97 | 0.97 |
| **2** | 0.97 | X | 0.99 |
| **3** | 0.97 | 0.99 | X |

## Intarreader intraclass correlation coefficients

| **Reader 1** | **Reader 2** | **Reader 3** |
| --- | --- | --- |
| 0.99 | 0.99 | 0.98 |

# Table S2. Comparison of patients characteristics between all patients converting to clinically apparent arthritis, those with serial MRIs and the final selection of RA patients studied.

|  | All converters  N=76 | |  | Converters with serial MR imaging  N=35 | |  | Patients diagnosed with RA & serial MR imaging  N=31 | |
| --- | --- | --- | --- | --- | --- | --- | --- | --- |
| Age in years, mean (SD) | 46 | (13) |  | 44 | (14) |  | 45 | (14) |
| Female sex, n (%) | 54 | (75) |  | 25 | (71) |  | 22 | (71) |
| Symptom duration in weeks, median (IQR) | 17 | (8–29) |  | 18 | (7–45) |  | 17 | (9-32) |
| Presence of morning stiffness ≥60 minutes, n (%) | 33 | (45) |  | 11 | (36) |  | 11 | (35) |
| 68-TJC, median (IQR) | 5 | (4–9) |  | 5 | (4–10) |  | 5 | (4–9) |
| Increased CRP (≥5 mg/L), n (%) | 20 | (30) |  | 6 | (17) |  | 6 | (19) |
| Autoantibody status |  |  |  |  |  |  |  |  |
| IgM-RF-positive (≥3.5 IU/mL), n (%) | 33 | (49) |  | 13 | (37) |  | 13 | (42) |
| ACPA-positive (≥7 U/mL), n (%) | 27 | (40) |  | 9 | (26) |  | 9 | (29) |

# Table S3. Patient characteristics of ACPA-positive and ACPA-negative patients studied, at presentation with CSA.

|  | ACPA-positive patients  N=9 | |  | ACPA-negative patients  N=22 | |
| --- | --- | --- | --- | --- | --- |
| Age in years, mean (SD) | 45 | (14) |  | 44 | (14) |
| Female sex, n (%) | 6 | (33) |  | 16 | (27) |
| Symptom duration in weeks, median (IQR) | 22 | (7–52) |  | 15 | (9–29) |
| Presence of morning stiffness ≥60 minutes, n (%) | 5 | (56) |  | 6 | (35) |
| 68-TJC, median (IQR) | 5 | (3–6) |  | 6 | (4–10) |

**Legend**:

Characteristics are in line with a previous report on differences in the symptomatic phase preceding ACPA-positive and ACPA-negative RA.[21]

**Table S4. Disease-modifying anti-rheumatic drugs prescribed when clinical arthritis was identified.**

| DMARD | N | (%) |
| --- | --- | --- |
| Methotrexate, n (%) | 19 | (61) |
| Sulfasalazine, n (%) | 4 | (13) |
| Hydroxychloroquine, n (%) | 8 | (26) |
| Prednisone in combination  with other cDMARD, n (%) | 9 | (29) |
| Certolizumab in setting  of clinical trial (C-EARLY), n (%) | 1 | (3) |

**Supplementary Methods. MRI scanning protocol and scoring**

*Detailed MR-scan protocol*

MR imaging was performed on an ONI MSK Extreme 1.5T extremity MR imaging system (GE Healthcare, Wisconsin, USA) using a 145mm coil for the foot and a 100mm coil for the hand. The patient was positioned in a chair beside the scanner, with the hand or foot fixed in the coil with cushions.

In the hand, the following sequences were acquired before contrast injection: T1-weighted fast spin-echo (FSE) sequence in the coronal plane (repetition time (TR) 575 ms, echo time (TE) 11.2 ms, acquisition matrix 388×288, echo train length (ETL) 2). After intravenous injection of gadolinium contrast (gadoteric acid, Guerbet, Paris, France, standard dose of 0.1 mmol/kg) the following sequences were obtained: T1-weighted FSE sequence with frequency selective fat saturation (fatsat) in the coronal plane (TR/TE 700/9.7ms, acquisition matrix 364×224, ETL 2), T1-weighted FSE sequence with frequency selective fat saturation in the axial plane (wrist: TR/TE 540/7.7 ms; acquisition matrix 320x192; ETL 2 and metacarpophalangeal joints: TR/TE 570/7.7 ms; acquisition matrix 320x192; ETL 2).

The obtained sequences of the forefoot were post-gadolinium sequences which included: T1-weighted FSE fatsat sequence in the axial plane (TR/TE 700/9.5ms; acquisition matrix 364x224, ETL 2) and: T1-weighted FSE fatsat sequence in the coronal plane (perpendicular to the axis of the metatarsals) (TR/TE 540/7.5ms; acquisition matrix 320x192, ETL 2). In the first 7 patients a T1-weighted sequence and a T2-weighted fat saturated sequence were acquired in the axial plane (relative to the anatomical position), before contrast agent administration at both time points. In 23 patients postcontrast, T1-weighted, fat saturated sequences were acquired in axial and coronal planes at both timepoints. This provided more information while reducing scanning-times. In one patient T1-weighted sequences and a T2-weighted fat saturated sequence were acquired in the axial plane (relative to the anatomical position), before contrast agent administration at presentation with arthralgia and postcontrast, T1-weighted, fat saturated sequences were acquired in axial and coronal planes at presentation with inflammatory arthritis. Analyses stratified per scanning protocol for the forefoot yielded similar results: with 62% of swollen joints having no prior subclinical inflammation in the first 8 patients (T1-weighted sequence and a T2-weighted fat saturated sequence of the forefoot at arthralgia) with and 71% of swollen joints having no prior subclinical inflammation in the remaining 27 patients (postcontrast, T1-weighted, fat saturated sequences of the forefoot).

Field-of-view was 100mm for the hand and 140mm for the foot. Coronal sequences of the hand had 18 slices with a slice thickness of 2mm and a slice gap of 0.2mm. Coronal sequences of the foot had 20 slices with a slice thickness of 3mm and a slice gap of 0.3mm. All axial sequences had a slice thickness of 3mm and a slice gap of 0.3mm with 20 slices for the wrist, 16 for the metacarpophalangeal joints and 14 for the foot.

According to the RAMRIS-method, T2-weighted fat suppressed sequences, or when this sequence is not available a short tau inversion recovery (STIR) sequence, should be used to assess Bone Marrow (O)Edema (BME). Previously, three studies have demonstrated that a contrast enhanced T1-weigthed fat suppressed sequence has a strong correlation with T2-weighted fat suppressed sequences.^1–3^ A T2-weighted image shows increased water signal and a contrast-enhanced T1-weighted sequence shows increased water content and the increased perfusion and interstitial leakage. A strong correlation has been shown in arthritis patients but also in patients without inflammatory diseases such as bone bruises, intraosseous ganglions, bone infarcts and even nonspecific cases.^2,3^ We used the contrast enhanced T1-weighted fat suppressed sequence as it allowed a shorter scan time and has a higher signal to noise ratio.

*MR scoring*

All bones, joints and tendons were scored semi-quantitatively. Similar to the RAMRIS method, synovitis score was scored based on the volume of enhancing tissue in the synovial compartment (none, mild, moderate, severe (range 0-3)). Similar to method described by Haavardsholm et al the tenosynovitis-score was based on the thickness of peritendinous effusion or synovial proliferation with contrast enhancement (normal, <2mm, 2-5mm, >5mm (range 0-3)).^4,5^ BME was depicted on a contrast enhanced T1-weigthed fat suppressed sequence and also scored on a 0-3 scale based on the affected volume of the bone (no BME, >0-33%, >33-66%, >66%). The scores of all joints were summed and the total BME, synovitis and tenosynovitis scored were summed as well, yielding the total MRI-inflammation-score.

**References**

1. Stomp W, Krabben A, Heijde D van der, *et al.* Aiming for a shorter rheumatoid arthritis MRI protocol: can contrast-enhanced MRI replace T2 for the detection of bone marrow oedema? *Eur Radiol* 2014;24:2614–22.

2. Schmid MR, Hodler J, Vienne P, *et al.* Bone Marrow Abnormalities of Foot and Ankle: STIR versus T1-weighted Contrast-enhanced Fat-suppressed Spin-Echo MR Imaging. *Radiology* 2002;224:463–9.

3. Mayerhoefer ME, Breitenseher MJ, Kramer J, *et al.* STIR vs. T1-weighted fat-suppressed gadolinium-enhanced MRI of bone marrow edema of the knee: Computer-assisted quantitative comparison and influence of injected contrast media volume and acquisition parameters. *J Magn Reson Imaging* 2005;22:788–93.

4. Østergaard M, Edmonds J, McQueen F, *et al.* An introduction to the EULAR–OMERACT rheumatoid arthritis MRI reference image atlas. *Ann Rheum Dis* 2005;64:i3–7.

5. Haavardsholm EA, Østergaard M, Ejbjerg BJ, *et al.* Introduction of a novel magnetic resonance imaging tenosynovitis score for rheumatoid arthritis: reliability in a multireader longitudinal study. *Ann Rheum Dis* 2007;66:1216–20.
